# The biopsychosocial-digital continuum of foot orthosis practice and research: the VALUATOR model

**DOI:** 10.1186/s13047-021-00468-6

**Published:** 2021-03-31

**Authors:** Kevin Deschamps, Chris Nester, Veronica Newton, Gabriel Gijon-Nogueron, Engin Simsek, Antoine Brabants

**Affiliations:** 1 Department of Podiatry, Artevelde University of Applied Sciences, Ghent, Belgium; 2 KULeuven- Department of Rehabilitation Sciences- Musculoskeletal Rehabilitation, Research Group, Campus Brugge, Spoorwegstraat 12, 8200 Brugge, Belgium; 3 Haute Ecole Leonard De Vinci, Institut D’Enseignement Supérieur Parnasse Deux-Alice, Division of Podiatry, Bruxelles, Belgium; 4 School of Health & Society, Brian Blatchford Building, Frederick Road Campus, University of Salford, Salford, M6 6PU UK; 5 Department of Nursing and Podiatry, University of Malaga, Malaga, Spain; 6 School of Physical Therapy and Rehabilitation Sciences, Dokuz Eylul University, İzmir, Turkey

## Abstract

Foot orthoses have been used for decades despite uncertainty surrunding their therapeutic efficacy. Orthoses have been used exclusively to affect neuro-biomechanical input and outcome variables, however, there is emerging evidence that therapeutic efficacy may be affected by a psychological stimulus. Critical appraisal of the literature highlights that there is no holistic model upon which foot orthosis practice is taught, practised nor investigated. This paper introduces a conceptual model of foot orthosis practice (**Valu**e B**a**sed Foo**t Orthosis** P**r**actice (VALUATOR) model) that embraces a broader range of factors that are pertinent to orthosis practice, incorporating contemporary health service behaviours and values into orthosis practice for the first time.

Within the VALUATOR model, foot orthosis design and clinical value is considered along a bio-psycho-social-digital continuum that reflects the reality of foot orthosis practice. The model contextualises the variable outcomes that are observed in research and practice within 6 key areas: 1) value, 2) person-centered approach, 3) zone of optimal bio-psycho-social stress, 4) bio-psycho-social assessment, 5) monitoring, 6) primary and secondary clinical strategies.

The VALUATOR model is targeted at students, lecturers, scientists and practitioners and includes carefully chosen terminology to support a robust basis for educational and scientific discussion. It is believed that it provides a contemporary viewpoint and a structured conceptual metaphor that builds on existing evidence from a wide range of sources, invites constructive intellectual debate, and is anchored in the experiences of practitioners too. Stress testing the VALUATOR model will help determine its model and support further developments and evolution of orthotic practice in a evidence based way.

## Background

Foot orthoses (FO) have been used for decades to treat foot and lower limb pathologies (e.g. knee and hip) [1–10]. Rigorous and expert analysis of the available evidence documenting the relative benefits and risks of foot orthosis therapy has led to the development of national and international practice guidelines [11–15]. The ultimate goal of these guidelines is to improve the effectiveness of care, optimize patient outcome and strive for the most cost-effective healthcare provision. To align practice with the available evidence, most of these guidelines adopted a therapeutic efficacy classification system, which typically comprises the following recommendations/hierarchy: 1) medically indicated and essential, 2) useful, 3) adjunctive, 4) not useful [11]. Ultimately, however, the decision to provide orthoses or not, occurs within the framework of evidence based practice (EBP) and involves the integration of the best available evidence with clinical knowledge and expertise, while considering patients’ unique needs and personal preferences.

Whilst practitioners are generally positive towards evidence based foot orthosis practice, considerable issues and uncertainties remain. For example, for what purpose or clinical endpoint are orthoses useful, how does cost and quality of the orthoses matter, and how do the nature and model of services that deliver orthoses impact outcomes [16–19]. Efficacy of foot orthoses has been subject to protracted debate and a number of factors associated with variable efficacy have been proposed [11, 19] including: i) the evolution of orthoses materials and associated technologies [20–23], ii) application by a wide range of practitioners [24, 25]; poor knowledge transfer between research and practice [26–28]; and increasing interest by consumers and other purchasers and dispensing by unqualified vendors [11].

Another factor that may contribute to the variable outcomes is a focus on the orthotic device as the key aspect of the intervention, and failure to contextualise orthoses within a wider person-centered approach. This is evident in some scientific studies/clinical trials where an association between an orthosis feature and biomechanical effect is assumed to be associated with the intended clinical, or personal, outcome. Some may criticize scientific studies for their use of prefabricated FO since customisation of a foot orthosis’ shape is central to many assumptions underlying orthosis theories. It is reasonable to assume that some degree of customization is required for a number of clinical conditions, especially where foot shape is affected or tissue viability impaired. However, research has demonstrated that there is not always a clear predictable difference between custom and non-custom foot orthoses in regards to clinical effectiveness [29].

The selection or customisation of orthoses is often driven exclusively by the need to affect neuro-biomechanical or biological input and outcome variables. For example, altering plantar pressure using a specific geometric orthotic feature, or using orthoses to stimulating plantar cutaneous receptors and thereafter affect postural control. This reflects an exclusively biological, or biophysical, basis to practice where defined inputs lead to defined outputs, and do so repeatedly. Most concepts underpinning foot orthosis practice assume orthoses provide a dose of a specific neuro-biomechanical stimulus that targets one or multiple neuro-biomechanical response(s). Well-known models and theories emerging from this dosage-response perspective include: 1) Tissue Stress Theory [30], 2) Zone of Optimal Stress theory (ZOOS) [31], 3) Root Model [32], 4) Rotational Equilibrium Theory of Foot Function [33], 5) The foot as sensory organ model [34, 35], to name a few. In addition, national guidelines may reflect this dominance of a biophysical definition of foot orthoses [11].

More recently, however, the potential for orthoses to achieve a therapeutic effect by providing a psychological as well biomechanical stimulus has started to receive attention [36]. In addition to softening of the strictly biological model, it is reasonable to assume that the design of foot orthoses and outcomes are influenced by factors such as the work or physical activity environment of the patient, the presence of kinesiophobia, the mental status of the patient, the type of pain, the absence of pain sensation, the patient’s (or payers) financial situation, to name a few. As a consequence, there is no holistic model upon which foot orthosis practice is taught, practised nor investigated. Encompassing these factors into paradigms of orthotic practice aligns well with the concept of ‘value based healthcare’ [37, 38], however, the latter has only been embraced in a very small number of countries (e.g. United Kingdom, United States, Germany). This paper aims, for the first time, to introduce a conceptual model of foot orthosis practice that embraces a broader range of factors that are pertinent in practice and reflect contemporary health service behaviours and values. The **Valu**e B**a**sed Foo**t Orthosis** P**r**actice (VALUATOR) model is targeted at students, lecturers, scientists and practitioners.

## The value based foot Orthosis practice (VALUATOR) model

**Fig. 1:**
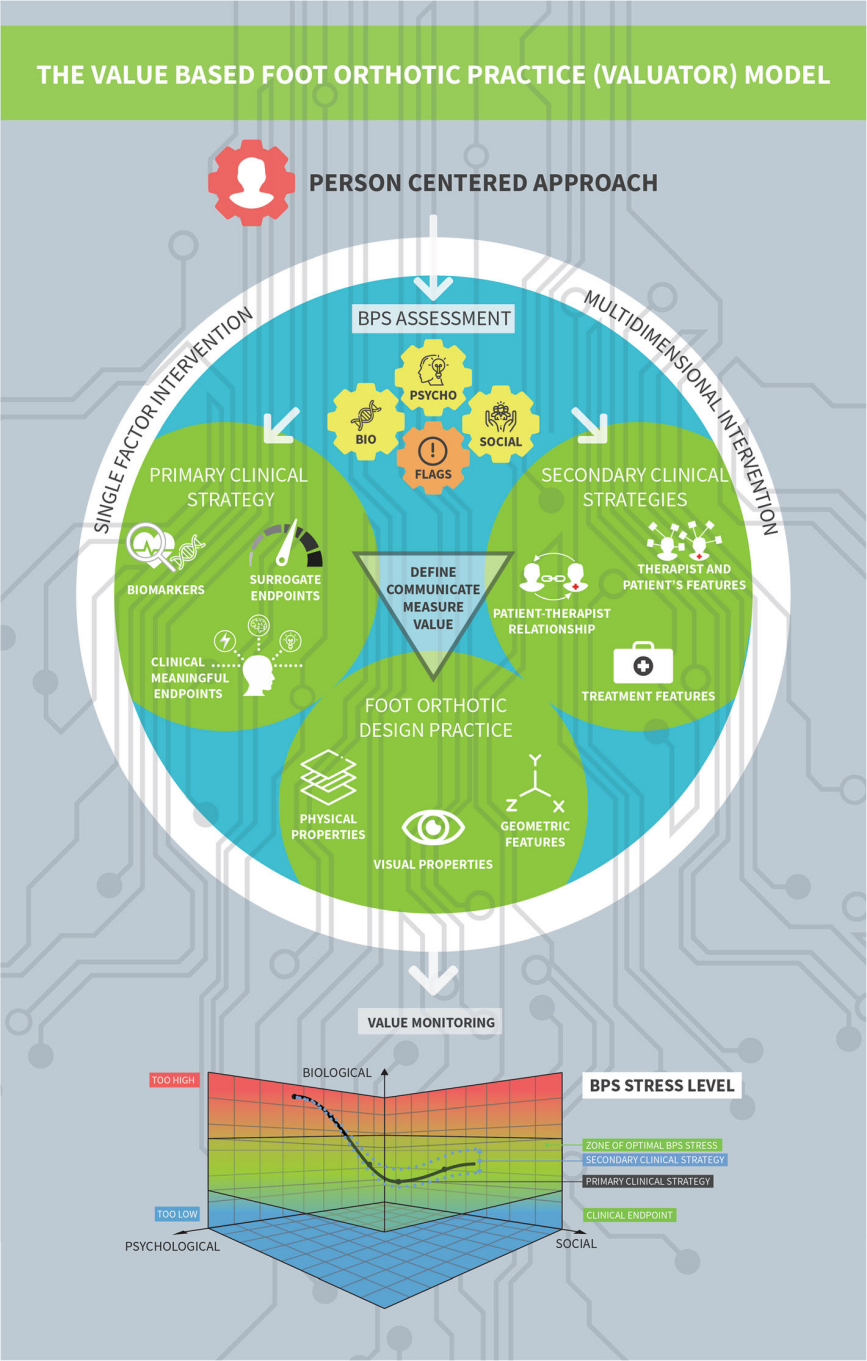
Conceptual map representing the six key areas of the VALUATOR model

We represent VALUATOR with a visual metaphor (Fig. 1) representing a value based healthcare approach [37, 39–41]. Within the model, foot orthosis design (i.e. the geometric and material properties of a foot orthosis) and clinical value is considered along a bio-psycho-social-digital continuum reflecting the nature of FO practice. The model helps to contextualise the variable outcomes that are observed in research and practice. The model is built upon 6 key areas that are discussed here below.

### A broad approach to VALUE

The concept of ‘value’ from health care comes in many forms and models of orthoses practice needs to reflect this. While there are often physical determinants of many musculoskeletal disorders, a growing body of evidence points towards a strong impact of psychological factors [42–45], mental health [46] and somatisation, particularly in chronic conditions [46, 47]. Furthermore, contrasts in cultural beliefs, behaviors and practices may contribute to major variation in the prevalence of certain disabling pain conditions globally, and several health professions routinely consider psychosocial factors alongside biological factors [47], so that value in all forms is fully appreciated.

Value is also affected by the changing and complex needs and expectations among different age populations. These factors affect outcomes and should therefore affect practice [26]. For example, athletes often set high (or unrealistic) expectations on how they will perform in competition, based on past results, competitors and coaching. A further example would be an obese person involved in a physical activity intervention programme who might require preventative measures to decrease the risk of musculoskeletal injuries as activity increases [48]. Health professionals providing orthoses are increasingly consulted in these situations, when there is no musculoskeletal pain or discomfort, and the value of practice focuses towards prevention and or performance rather than treatment.

Most people are also consumers and these experiences strongly shape how we might value our experiences of health care delivery, accessing what is needed, when desired and at speed. Together with the digital (r)evolution that medical and allied health professions are facing, some underpinned by consumer trends (e.g. online services), there has perhaps been an evolution of the components of the health professional -patient relationship that are valued [49, 50]. Consequently, a conceptual model of foot orthosis practice must involve qualitative, integrated and holistic approaches to care so that value is identified and incorporated wherever possible, reflecting the specific care needs and/or support requested by a particular person.

### Person-centered goal setting and measuring value

In addition to a BPS contextualisation of a health care and/or support question, there is an emerging body of evidence demonstrating that person-centered goal setting is the gold standard approach for a number of reasons [51, 52]. First, research has shown that it is important for clinicians to involve a person in goal setting and to respect the values and preferences of a person [38]. Second, such goal setting might improve allocation of resources. Finally, structured goal setting can result in higher levels of motivation, self-efficacy and health-related quality of life [53].

Although the benefits of patient-centered goal setting are widely recognized, implementation in research practice remains challenging. Too often, so-called surrogate measures are selected by the researcher or professional as a substitute for clinical meaningful endpoints. With respect to foot orthosis practice, these goals are often based on ‘biological endpoints’ (e.g. plantar pressure, changes in foot kinematics, etc), when these surrogate measures have yet to be validated as being associated with clinical outcomes. Moreover, clinicians often operate in the context of a multidimensional health care and/or support question and therefore also in the context of multidimensional interventions. The contributions of other interventions, and their interactions with orthosis use, is often underestimated and not explicitly recognized in current foot orthosis practice models.

The VALUATOR model aims to tackle the reductionist viewpoint by promoting goal-setting theory and monitoring value across a three-domain classification system [54–56]. Thinking about and setting goals may be difficult for some and professionals need the skills to assist patients in this process [57]. Proposed whole-person body domains can be used for goal setting, and encompass physical, ethical, cultural, family, financial, psychological, social, spiritual and global domains [57, 58]. More details about the three domain classification system for monitoring value is provided further in the text.

### Zone of optimal bio-psycho-social stress (ZOOBPSS)

The VALUATOR model of orthoses practice projects a given person along/within a BPS stress continuum and each individual has a Zone of Optimal Bio-Psycho-Social Stress (ZOOBPSS). This is a zone in which the person experiences body, mindand tissue homeostasis (optimal function). The boundaries of this zone have no definitive cut-off, are dynamic over time and are highly person specific, hence, the therapist aims to estimate these boundaries through a BPS assessment. In the most favorable clinical situation, a professional is able to provide a robust estimation about the upper and lower boundaries of the ZOOBPSS, but this is rarely the case in relation to complex interventions such as orthosis therapy (Fig. 1). The concept of ZOOBPS extends into a more person centred domain the more strictly biological concepts, the ‘Zone of Optimal Stress’ being one example [31]. The latter focused on the physical forces affecting living cells and tissues and their association with foot related complaints, and ignores the evidence for psychological and social factors affecting a health care and/or support question as well as outcomes of interventions [46].

### BPS assessment

The emerging evidence for the association of psychosocial factors with musculoskeletal disorders demands their integration with existing clinical assessment and orthotic skills. The VALUATOR model invites integration of numerous clinical biopsychosocial assessments with practice, to understand and explain the mechanism and/or motivation for a health care (and/or support) question presented to a health professional and thereby guide clinical management. By advocating any of a number of the many assessments available, the model asks the professional to first understand what value is being sought by the patient and selecting assessment accordingly. An example of such a clinical assessment model is the PSCEBSM (Pain-Somatic-Cognition-Emotional-Behavioral-Social-Motivation) model, by Wijema et al. (2016) [48]. A plethora of tools and questionnaires have been developed (e.g. Foot Function Index, Leeds Foot Impact Scale, Widespread Pain Index, Visual Analogue Scale, Central Sensitization Inventory, Pain Catastrophizing Scale, Tampa-Scale of Kinesiophobia, Psychology Inflexibility in Pain Scale, etc) in order to provide clinical meaningful (valuable) measures when conducting clinical biopsychosocial assessments.

### Monitoring value

Porter (2010) proposed measurement of outcomes important to individuals and assessment of costs using time-driven activity-based costing (so-called Value-Based Health Care) [37]. The VALUATOR model is an interpretation of that approach, specific to orthosis practice. In an attempt to encourage a value based health care approach in foot orthosis practice, professionals may encounter considerable challenges when selecting valuable endpoints or measures. To assist users of the VALUATOR model, the following three-domain classification system has been selected for its strong link with evidence based practice: (1) clinical measures, (2) non-clinical measures, and (3) surrogate measures [59].

Clinically meaningful measures/endpoints represent outcome measures about how a person feels, functions or survives. These may be measured objectively or subjectively, and are either (i) based upon judgements or interpretations of clinical signs by the professional, (ii) reported by patients (so-called patient reported outcome measures, PROMS) or (iii) observer-reported, such as a parents’ feedback about daily activity level of a child [59].

Non-clinical measures/endpoints, including biomarkers, are objectively measured indicators of a physiologic or pathogenic process, for example the mechanobiological response at the level of the skin, tendon, muscle, cartilage and bones [60, 61].

Surrogate measures are those outcomes which are closely associated with a clinically meaningful endpoint and which may serve as a substitute [59].

Outcome measures and surrogate measures have been categorized according to their scientific validation. Level 1 is a true clinical meaningful measure; Level 2 is a validated surrogate; Level 3 is a non-validated surrogate that is ‘reasonably likely to predict clinical benefit’, and Level 4 represents merely a correlational measure of biological activity [62]. The user of the VALUATOR model is encouraged to adopt this three-domain classification system since it will help in determining gaps and weaknesses in foot orthosis research, but equally important, help in better appreciating the socio-economic value of foot orthoses.

### Clinical strategies and foot orthosis design

The VALUATOR models encourages professionals to adopt multidimensional BPS dosage-response modelling and demands a broader perspective on factors affecting the relationship between professional practice and patient-centered outcomes. It is suggested to distinguish between primary and secondary clinical strategies [59]. The former focuses on clinical perspectives associated with the clinical, non-clinical and surrogate measures detailed above. The latter invites consideration of promising additional clinical strategies which may influence the foot orthosis design process (dosage), and ultimately, the clinical outcome and value (response).

#### Primary clinical strategies

There is a vast body of evidence addressing the impact of foot orthoses on clinical and surrogate measures [63], although evidence for changes in endpoints due to specific changes in orthosis design is sparce [64]. The VALUATOR puts forward three pillars in foot orthotic design practice: 1) geometric features, 2) material properties and 3) visual properties. The primary clinical strategy requires that orthotic design is aligned with / or guided by pre-established BPS clinical meaningful endpoints and surrogate measures. Thus in contemporary practice, selection of specific foot orthosis features, material properties and visual properties is guided by the presence or absence of kinesiophobia, patients’ expectations, plantar pressure benchmarks, sociological measures, biophysical characteristics of skin lesions, to name a few.

#### Secondary clinical strategies

There are a wide range of behavioural, environmental and psychosocial factors that can become part of clinical strategies and affect orthotic outcomes. The management of placebo and avoidance of nocebo responses have recently been suggested as promising additional clinical strategies [65] and are a platform for considering many of these factors. The administration of FO is a multidimensional therapeutic ritual that engages neurobiological mechanisms behind placebo and nocebo effects, explained by either a ‘pain’ or ‘motor performance’ model [65]. Clinical strategies that may enhance these effects can be broken down into three domains [65], namely features of the (1) therapist and patient, (2) the patient-therapist relationship, and (3) the treatment. Those which are most closely related to foot orthosis practice include:

- Communicating the clinical objectives of orthosis practice including the functional effects or the overall feeling that one may expect when wearing the foot orthoses.
- Patient-centred approach, with an opportunity to select a specific material or visual aspects of an orthosis (e.g. colour).
- Environmental setting, including use of apparently “state-of-the art” technologies (e.g. 3D scanners, pressure analysis) that impact on the perception of expertise, professionalism and value, which in turn, may affect the patients view, expectations and response (e.g. better adherence).
- Psychophysical factors of the orthosis including the value placed on sensations and perceived comfort. Evidence from Enclothed Cognition Theory suggests that physical and visual properties may influence human performance [66–68].

## Discussion and conclusion

The aim of the current manuscript was to introduce a conceptual model of foot orthosis practice which may be used by students, lecturers, scientists and therapists around the globe. The model uses a visual metaphor to introduce a value based healthcare approach which projects orthotic practice and the clinical value of foot orthoses along a bio-psycho-social-digital continuum.

The biological component of the VALUATOR model assumes that clinical symptoms are the result of loads acting upon foot tissues and that orthoses adjust these loads and thereby affect symptoms [63]. Adjusting loads applied to the foot is a complex phenomenon not least because the foot orthosis has four functional surfaces [63] and the foot, orthosis and footwear have different static and dynamic properties. The priority is often the upper orthotic surface that contacts the foot and the extent to which it should reflect the static shape of the foot has been the topic of much debate. The two sides of the orthosis interact with the shoe upper, and the base with the shoe sole. It is through these three sides that forces are transferred through the shoe to the foot, and thus all four sides affect the effect of the orthosis. of the four sides of an orthosis are rarely debated thus far and this points at the need for work on this respect to improve the biological components in the model.

That said, the association between specific orthotic features and predictable biomechanical responses (a dose – response model) and outcomes of value is unproven. This is unlikely given the multi factorial nature of most foot problems. Indeed, evidence points not to specific orthosis design features having a predictable outcome, but rather for specific effects (e.g. pressure reduction) achieving clinical aims (reduced ulceration risk), regardless of the orthosis feature used to achieve it. Whilst a biomechanical lens has thus far dominated the adoption of a dosage-response model in orthotic practice, the VALUATOR model goes beyond this and proposes a broader view that better reflects the evidence and practical experience too.

The digital component of the bio-psycho-social-digital continuum has only been moderately illustrated so far. The factors which influence the process of foot shape capture (e.g. plaster of paris, foam impression box, digital scan), orthoses design (shape and material choices, specified in digital design software or by written prescription) and orthosis manufacture (hand-made versus computer aided manufacture) vary across professional disciplines, industry, educational and research contexts. Debates in the literature compare these digital and manual processes, focussing on levels of accuracy and repeatability, but this assumes these are critical to the achievement of the intended outcomes and thus deriving value. This is unproven and whilst it is reasonable to assume that digital workflows will support transition of orthotic practice from artisan art to a clinical speciality with appropriate medical device production quality control [22], much of this requires further research.

The VALUATOR model embraces the three-domain classification system described by McLeod et al. [59]. Using this highlights the fact that current foot orthosis practice often relies upon surrogate measures, and too much reliance on these is an ongoing risk. By contrast, greater focus on biomarkers might facilitate greater understanding of mechanobiology and foot pathology. This also reflects the fact that by evolving the conceptional basis of orthosis practice through greater incorporation of non-biological elements, we are not diminishing their importance. Rather, we are seeking to contextualise them in the context of other evidence and practice.

The majority of the quality measures identified as examples within VALUATOR have not yet been validated in the context of foot related disorders. This raises questions about the viability of the proposed model, although at this initial stage, the conceptual basis for the VALUATOR model is not sensitive to the specific measures it advocates. This also reflects the fact that any model is a platform for posing unanswered questions that research should seek to answer, and thereby allow concepts and practical actions that follow to evolve as increasingly evidence based. Recognising the model as incomplete, needing further work and as a dynamic knowledge base for professional practice, is in itself an evolution from previous models, which have tended to provide conclusive answers to questions about orthotic practice, regardless of the actual evidence underpinning the ideas.

In conclusion, a value based foot orthosis practice model has been proposed which integrates different concepts, viewpoints and quality measures. Whatever promise the current model holds, it is believed that it provides a contemporary viewpoint and a structured conceptual metaphor to support the intellectual debate and teaching among the professions that use foot orthoses.

## Data Availability

Not applicable.
